# Ethyl 4-[(4-chloro­phen­oxy)meth­yl]-2-(4-nitro­phen­yl)-1,3-thia­zole-5-carboxyl­ate

**DOI:** 10.1107/S1600536811049774

**Published:** 2011-11-25

**Authors:** Zhi-Rong Deng, Shu-Qing Wang, Wei-Li Dong, Run-Ling Wang

**Affiliations:** a Tianjin Key Laboratory on Technologies Enabling Development of Clinical Therapeutics and Diagnostics (Theranostics), School of Pharmacy, Tianjin Medical University, Tianjin 300070, People’s Republic of China

## Abstract

The title compound, C_19_H_15_ClN_2_O_5_S, contains two mol­ecules (*A* and *B*) in the asymmetric unit. In mol­ecule *A*, the dihedral angles between the thia­zole ring and the pendant chloro­benzene and nitro­benzene rings are 72.14 (15) and 3.03 (15)°, respectively. The corresponding angles for mol­ecule *B* are 45.56 (16) and 1.51 (14)°, respectively. In the crystal, both mol­ecules form inversion dimers linked by pairs of weak C—H⋯O inter­actions.

## Related literature

For the biological activity of related compounds and for related structures, see: Liu *et al.* (2011*a* ▶,*b* ▶,*c* ▶,*d* ▶). For further synthetic details, see: Cho *et al.* (2010 ▶).
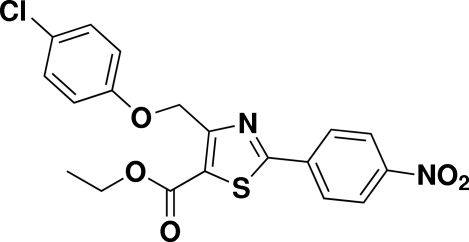


## Experimental

### 

#### Crystal data

C_19_H_15_ClN_2_O_5_S
*M* *_r_* = 418.84
Triclinic, 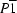

*a* = 7.658 (2) Å
*b* = 7.736 (2) Å
*c* = 31.462 (9) Å
α = 95.414 (8)°
β = 93.595 (13)°
γ = 95.536 (9)°
*V* = 1841.9 (9) Å^3^
*Z* = 4
Mo *K*α radiation
μ = 0.36 mm^−1^
*T* = 113 K
0.20 × 0.18 × 0.10 mm

#### Data collection

Rigaku Saturn724+ CCD diffractometer
Absorption correction: multi-scan (*CrystalClear*; Rigaku/MSC, 2005 ▶) *T*_min_ = 0.932, *T*_max_ = 0.965
16159 measured reflections
6636 independent reflections
4821 reflections with *I* > 2σ(*I*)
*R*_int_ = 0.054

#### Refinement

*R*[*F* ^2^ > 2σ(*F* ^2^)] = 0.058
*wR*(*F* ^2^) = 0.138
*S* = 1.07
6636 reflections
507 parameters
H-atom parameters constrained
Δρ_max_ = 0.70 e Å^−3^
Δρ_min_ = −0.54 e Å^−3^

### 

Data collection: *CrystalClear* (Rigaku/MSC, 2005 ▶); cell refinement: *CrystalClear*; data reduction: *CrystalClear*; program(s) used to solve structure: *SHELXS97* (Sheldrick, 2008 ▶); program(s) used to refine structure: *SHELXL97* (Sheldrick, 2008 ▶); molecular graphics: *SHELXTL* (Sheldrick, 2008 ▶); software used to prepare material for publication: *SHELXTL*.

## Supplementary Material

Crystal structure: contains datablock(s) global, I. DOI: 10.1107/S1600536811049774/hb6513sup1.cif

Structure factors: contains datablock(s) I. DOI: 10.1107/S1600536811049774/hb6513Isup2.hkl

Supplementary material file. DOI: 10.1107/S1600536811049774/hb6513Isup3.cml

Additional supplementary materials:  crystallographic information; 3D view; checkCIF report

## Figures and Tables

**Table 1:** Hydrogen-bond geometry (Å, °)

*D*—H⋯*A*	*D*—H	H⋯*A*	*D*⋯*A*	*D*—H⋯*A*
C1—H1⋯O5^i^	0.95	2.37	3.314 (4)	172
C20—H20⋯O10^ii^	0.95	2.50	3.450 (4)	173

Symmetry codes: (i) 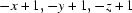; (ii) 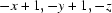.

**Fig. 1.**
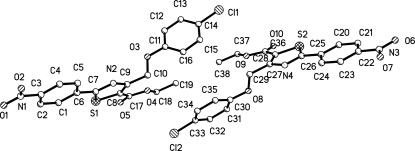
The molecular structure of (I). Displacement ellipsoids are drawn at the 30% probability level.

## supplementary crystallographic information

### Experimental

The title compound was prepared according to the literature procedures (Cho *et al.*, 2010). Colourless blocks of (I) were grown from slow evaporation of ethanol solution at room temperature.

### Refinement

All the H atoms were positioned geometrically (C—H = 0.93–0.97 Å) and refined as riding with *U*_iso_(H) = 1.2*U*_eq_(C) or 1.5*U*_eq_(methyl C).

### Crystal data

**Table:**

C_19_H_15_ClN_2_O_5_S	*Z* = 4
*M**_r_* = 418.84	*F*(000) = 864
Triclinic, *P*1	*D*_x_ = 1.510 Mg m^−^^3^
*a* = 7.658 (2) Å	Mo *K*α radiation, λ = 0.71073 Å
*b* = 7.736 (2) Å	Cell parameters from 5373 reflections
*c* = 31.462 (9) Å	θ = 2.0–28.0°
α = 95.414 (8)°	µ = 0.36 mm^−^^1^
β = 93.595 (13)°	*T* = 113 K
γ = 95.536 (9)°	Block, colorless
*V* = 1841.9 (9) Å^3^	0.20 × 0.18 × 0.10 mm

### Data collection

**Table:**

Rigaku Saturn724+ CCD diffractometer	6636 independent reflections
Radiation source: fine-focus sealed tube	4821 reflections with *I* > 2σ(*I*)
graphite	*R*_int_ = 0.054
Detector resolution: 28.5714 pixels mm^-1^	θ_max_ = 25.3°, θ_min_ = 2.0°
profile data from ω–scans	*h* = −9→9
Absorption correction: multi-scan (*CrystalClear*; Rigaku/MSC, 2005)	*k* = −9→9
*T*_min_ = 0.932, *T*_max_ = 0.965	*l* = −34→37
16159 measured reflections	

### Refinement

**Table:**

Refinement on *F*^2^	Primary atom site location: structure-invariant direct methods
Least-squares matrix: full	Secondary atom site location: difference Fourier map
*R*[*F*^2^ > 2σ(*F*^2^)] = 0.058	Hydrogen site location: inferred from neighbouring sites
*wR*(*F*^2^) = 0.138	H-atom parameters constrained
*S* = 1.07	*w* = 1/[σ^2^(*F*_o_^2^) + (0.0487*P*)^2^] where *P* = (*F*_o_^2^ + 2*F*_c_^2^)/3
6636 reflections	(Δ/σ)_max_ < 0.001
507 parameters	Δρ_max_ = 0.70 e Å^−^^3^
0 restraints	Δρ_min_ = −0.54 e Å^−^^3^

### Special details

**Table:**

Geometry. All e.s.d.'s (except the e.s.d. in the dihedral angle between two l.s. planes) are estimated using the full covariance matrix. The cell e.s.d.'s are taken into account individually in the estimation of e.s.d.'s in distances, angles and torsion angles; correlations between e.s.d.'s in cell parameters are only used when they are defined by crystal symmetry. An approximate (isotropic) treatment of cell e.s.d.'s is used for estimating e.s.d.'s involving l.s. planes.
Refinement. Refinement of *F*^2^ against ALL reflections. The weighted *R*-factor *wR* and goodness of fit *S* are based on *F*^2^, conventional *R*-factors *R* are based on *F*, with *F* set to zero for negative *F*^2^. The threshold expression of *F*^2^ > σ(*F*^2^) is used only for calculating *R*-factors(gt) *etc*. and is not relevant to the choice of reflections for refinement. *R*-factors based on *F*^2^ are statistically about twice as large as those based on *F*, and *R*- factors based on ALL data will be even larger.

### Fractional atomic coordinates and isotropic or equivalent isotropic displacement parameters (Å^2^)

**Table:**

	*x*	*y*	*z*	*U*_iso_*/*U*_eq_	
Cl1	0.06288 (14)	0.22346 (16)	0.14799 (3)	0.0624 (3)	
Cl2	0.75805 (14)	0.89132 (14)	0.35049 (3)	0.0543 (3)	
S1	0.31213 (10)	0.60383 (11)	0.47252 (2)	0.0282 (2)	
S2	0.38418 (10)	0.69544 (11)	0.02723 (2)	0.0270 (2)	
O1	−0.2945 (3)	0.9265 (4)	0.62204 (8)	0.0494 (7)	
O2	−0.4697 (3)	0.9946 (3)	0.57059 (8)	0.0458 (7)	
O3	0.1342 (3)	0.4482 (3)	0.33207 (7)	0.0371 (6)	
O4	0.5159 (3)	0.4840 (3)	0.36414 (7)	0.0329 (6)	
O5	0.5999 (3)	0.4344 (4)	0.43078 (7)	0.0481 (7)	
O6	0.0810 (3)	1.2838 (4)	−0.12206 (7)	0.0473 (7)	
O7	0.0164 (3)	1.4680 (3)	−0.07058 (7)	0.0431 (6)	
O8	0.5491 (3)	0.8696 (3)	0.16726 (7)	0.0391 (6)	
O9	0.4837 (3)	0.4928 (3)	0.13384 (7)	0.0393 (6)	
O10	0.5477 (3)	0.4164 (3)	0.06707 (7)	0.0479 (7)	
N1	−0.3375 (4)	0.9339 (4)	0.58378 (9)	0.0351 (7)	
N2	0.0807 (3)	0.6965 (4)	0.41884 (8)	0.0284 (6)	
N3	0.0757 (3)	1.3317 (4)	−0.08384 (9)	0.0329 (7)	
N4	0.3024 (3)	0.9408 (4)	0.08080 (8)	0.0269 (6)	
C1	0.0473 (4)	0.7472 (4)	0.53661 (10)	0.0273 (8)	
H1	0.1548	0.7055	0.5458	0.033*	
C2	−0.0636 (4)	0.8068 (4)	0.56656 (10)	0.0292 (8)	
H2	−0.0337	0.8070	0.5963	0.035*	
C3	−0.2193 (4)	0.8660 (4)	0.55205 (10)	0.0283 (8)	
C4	−0.2677 (4)	0.8684 (4)	0.50915 (10)	0.0264 (7)	
H4	−0.3748	0.9112	0.5002	0.032*	
C5	−0.1574 (4)	0.8076 (4)	0.47948 (10)	0.0264 (8)	
H5	−0.1897	0.8060	0.4498	0.032*	
C6	0.0014 (4)	0.7483 (4)	0.49283 (9)	0.0258 (7)	
C7	0.1180 (4)	0.6887 (4)	0.45996 (10)	0.0257 (8)	
C8	0.3452 (4)	0.5764 (4)	0.41898 (9)	0.0265 (8)	
C9	0.2093 (4)	0.6326 (4)	0.39547 (10)	0.0274 (8)	
C10	0.1867 (4)	0.6278 (5)	0.34789 (9)	0.0337 (8)	
H10A	0.0954	0.7027	0.3394	0.040*	
H10B	0.2985	0.6696	0.3362	0.040*	
C11	0.1246 (4)	0.4053 (5)	0.28864 (10)	0.0325 (8)	
C12	0.0871 (4)	0.2302 (5)	0.27464 (11)	0.0414 (9)	
H12	0.0740	0.1475	0.2950	0.050*	
C13	0.0684 (4)	0.1737 (5)	0.23175 (11)	0.0435 (10)	
H13	0.0434	0.0529	0.2224	0.052*	
C14	0.0866 (4)	0.2951 (6)	0.20225 (11)	0.0419 (10)	
C15	0.1222 (5)	0.4678 (5)	0.21532 (11)	0.0448 (10)	
H15	0.1323	0.5494	0.1946	0.054*	
C16	0.1441 (4)	0.5272 (5)	0.25884 (11)	0.0400 (9)	
H16	0.1717	0.6479	0.2680	0.048*	
C17	0.5000 (4)	0.4921 (5)	0.40608 (11)	0.0304 (8)	
C18	0.6616 (4)	0.3931 (5)	0.34835 (11)	0.0395 (9)	
H18A	0.6565	0.2742	0.3577	0.047*	
H18B	0.7758	0.4579	0.3593	0.047*	
C19	0.6409 (4)	0.3845 (5)	0.30047 (10)	0.0427 (10)	
H19A	0.5281	0.3186	0.2901	0.064*	
H19B	0.7370	0.3262	0.2882	0.064*	
H19C	0.6440	0.5030	0.2918	0.064*	
C20	0.2591 (4)	0.9569 (4)	−0.03709 (9)	0.0254 (7)	
H20	0.3027	0.8497	−0.0463	0.030*	
C21	0.2033 (4)	1.0641 (4)	−0.06709 (10)	0.0253 (7)	
H21	0.2087	1.0325	−0.0969	0.030*	
C22	0.1395 (4)	1.2188 (4)	−0.05227 (10)	0.0263 (8)	
C23	0.1314 (4)	1.2715 (4)	−0.00937 (9)	0.0260 (7)	
H23	0.0880	1.3790	−0.0004	0.031*	
C24	0.1879 (4)	1.1641 (4)	0.02008 (10)	0.0258 (7)	
H24	0.1835	1.1980	0.0498	0.031*	
C25	0.2512 (3)	1.0067 (4)	0.00690 (9)	0.0215 (7)	
C26	0.3069 (3)	0.8944 (4)	0.03974 (9)	0.0227 (7)	
C27	0.4094 (4)	0.6720 (4)	0.08080 (9)	0.0256 (7)	
C28	0.3598 (4)	0.8142 (4)	0.10414 (9)	0.0260 (8)	
C29	0.3674 (4)	0.8490 (5)	0.15229 (10)	0.0352 (9)	
H29A	0.3119	0.9563	0.1607	0.042*	
H29B	0.3043	0.7502	0.1647	0.042*	
C30	0.5855 (4)	0.8708 (5)	0.21057 (10)	0.0337 (9)	
C31	0.7463 (4)	0.8208 (5)	0.22347 (11)	0.0419 (10)	
H31	0.8221	0.7818	0.2026	0.050*	
C32	0.7992 (5)	0.8266 (5)	0.26642 (11)	0.0437 (10)	
H32	0.9109	0.7929	0.2751	0.052*	
C33	0.6870 (5)	0.8826 (5)	0.29680 (10)	0.0385 (9)	
C34	0.5246 (4)	0.9283 (5)	0.28490 (11)	0.0379 (9)	
H34	0.4479	0.9636	0.3059	0.045*	
C35	0.4729 (5)	0.9223 (5)	0.24120 (11)	0.0386 (9)	
H35	0.3602	0.9537	0.2325	0.046*	
C36	0.4860 (4)	0.5145 (5)	0.09238 (11)	0.0317 (8)	
C37	0.5723 (6)	0.3412 (5)	0.14607 (12)	0.0566 (12)	
H37A	0.5024	0.2315	0.1338	0.068*	
H37B	0.6898	0.3444	0.1345	0.068*	
C38	0.5903 (6)	0.3461 (6)	0.19137 (12)	0.0651 (13)	
H38A	0.6576	0.4558	0.2034	0.098*	
H38B	0.6522	0.2476	0.1994	0.098*	
H38C	0.4735	0.3381	0.2025	0.098*	

### Atomic displacement parameters (Å^2^)

**Table:**

	*U*^11^	*U*^22^	*U*^33^	*U*^12^	*U*^13^	*U*^23^
Cl1	0.0789 (8)	0.0728 (9)	0.0332 (6)	0.0111 (6)	0.0073 (5)	−0.0124 (5)
Cl2	0.0793 (7)	0.0543 (7)	0.0292 (5)	0.0136 (6)	−0.0041 (5)	0.0023 (5)
S1	0.0269 (4)	0.0314 (5)	0.0265 (5)	0.0083 (4)	0.0003 (3)	0.0002 (4)
S2	0.0299 (4)	0.0238 (5)	0.0267 (5)	0.0065 (4)	−0.0012 (3)	−0.0019 (4)
O1	0.0576 (17)	0.063 (2)	0.0317 (15)	0.0229 (14)	0.0142 (12)	0.0032 (13)
O2	0.0411 (15)	0.0510 (19)	0.0493 (16)	0.0228 (13)	0.0121 (12)	0.0010 (13)
O3	0.0433 (14)	0.0385 (16)	0.0268 (13)	−0.0016 (12)	0.0015 (10)	−0.0046 (11)
O4	0.0287 (12)	0.0390 (16)	0.0329 (14)	0.0123 (11)	0.0078 (10)	0.0001 (11)
O5	0.0421 (15)	0.071 (2)	0.0341 (14)	0.0317 (14)	−0.0020 (11)	0.0000 (14)
O6	0.0652 (17)	0.0533 (19)	0.0289 (14)	0.0219 (14)	0.0086 (12)	0.0126 (13)
O7	0.0471 (15)	0.0401 (17)	0.0462 (16)	0.0212 (13)	0.0030 (12)	0.0088 (13)
O8	0.0378 (14)	0.0485 (18)	0.0278 (13)	−0.0048 (12)	−0.0015 (10)	−0.0016 (12)
O9	0.0582 (16)	0.0258 (15)	0.0341 (14)	0.0139 (12)	−0.0096 (11)	0.0026 (11)
O10	0.0687 (18)	0.0373 (17)	0.0391 (15)	0.0269 (14)	−0.0019 (13)	−0.0062 (12)
N1	0.0354 (17)	0.0311 (19)	0.0399 (19)	0.0074 (14)	0.0118 (14)	−0.0004 (15)
N2	0.0306 (15)	0.0284 (17)	0.0264 (15)	0.0072 (12)	0.0024 (12)	−0.0004 (12)
N3	0.0274 (15)	0.038 (2)	0.0357 (18)	0.0065 (14)	0.0037 (12)	0.0100 (14)
N4	0.0283 (15)	0.0282 (17)	0.0250 (15)	0.0064 (12)	0.0047 (11)	0.0014 (12)
C1	0.0223 (16)	0.029 (2)	0.0310 (19)	0.0076 (14)	−0.0013 (13)	0.0005 (15)
C2	0.0328 (18)	0.028 (2)	0.0263 (18)	0.0046 (15)	0.0030 (14)	0.0008 (15)
C3	0.0315 (18)	0.0208 (19)	0.0336 (19)	0.0064 (15)	0.0109 (14)	−0.0009 (15)
C4	0.0234 (17)	0.0211 (19)	0.0341 (19)	0.0030 (14)	0.0013 (14)	−0.0001 (15)
C5	0.0274 (17)	0.024 (2)	0.0271 (18)	0.0039 (14)	0.0002 (13)	−0.0004 (14)
C6	0.0298 (17)	0.0201 (19)	0.0271 (18)	0.0032 (14)	0.0014 (13)	0.0009 (14)
C7	0.0242 (17)	0.0207 (19)	0.0312 (19)	0.0023 (14)	−0.0015 (13)	0.0001 (15)
C8	0.0281 (17)	0.023 (2)	0.0272 (18)	0.0034 (14)	0.0018 (13)	−0.0023 (15)
C9	0.0305 (18)	0.029 (2)	0.0237 (17)	0.0053 (15)	0.0063 (13)	0.0010 (15)
C10	0.0356 (19)	0.038 (2)	0.0285 (19)	0.0097 (17)	0.0048 (14)	−0.0002 (16)
C11	0.0260 (18)	0.044 (2)	0.0272 (19)	0.0087 (16)	0.0039 (14)	−0.0024 (17)
C12	0.046 (2)	0.038 (3)	0.039 (2)	−0.0013 (18)	0.0043 (17)	0.0000 (18)
C13	0.041 (2)	0.042 (3)	0.045 (2)	0.0008 (19)	0.0050 (17)	−0.005 (2)
C14	0.039 (2)	0.055 (3)	0.032 (2)	0.0090 (19)	0.0072 (16)	−0.0057 (19)
C15	0.057 (2)	0.044 (3)	0.036 (2)	0.013 (2)	0.0135 (18)	0.0045 (19)
C16	0.044 (2)	0.044 (3)	0.035 (2)	0.0099 (18)	0.0080 (16)	0.0050 (18)
C17	0.0273 (18)	0.028 (2)	0.035 (2)	0.0039 (15)	0.0023 (15)	−0.0031 (16)
C18	0.0293 (19)	0.044 (3)	0.047 (2)	0.0147 (17)	0.0132 (16)	−0.0025 (18)
C19	0.048 (2)	0.040 (3)	0.042 (2)	0.0097 (18)	0.0189 (17)	0.0019 (18)
C20	0.0247 (17)	0.024 (2)	0.0281 (18)	0.0072 (14)	0.0024 (13)	0.0014 (14)
C21	0.0236 (16)	0.025 (2)	0.0256 (18)	0.0000 (14)	0.0024 (13)	−0.0012 (14)
C22	0.0198 (16)	0.029 (2)	0.0313 (19)	0.0034 (14)	0.0002 (13)	0.0070 (15)
C23	0.0197 (16)	0.026 (2)	0.0318 (19)	0.0017 (14)	0.0022 (13)	0.0004 (15)
C24	0.0228 (16)	0.030 (2)	0.0239 (17)	0.0051 (14)	0.0022 (13)	−0.0019 (15)
C25	0.0164 (15)	0.0236 (19)	0.0235 (17)	−0.0007 (13)	0.0004 (12)	0.0011 (14)
C26	0.0178 (16)	0.0202 (19)	0.0291 (18)	0.0002 (13)	0.0008 (13)	−0.0008 (14)
C27	0.0251 (17)	0.028 (2)	0.0241 (17)	0.0036 (14)	−0.0020 (13)	0.0046 (14)
C28	0.0245 (17)	0.029 (2)	0.0242 (17)	0.0018 (15)	−0.0003 (13)	0.0019 (15)
C29	0.038 (2)	0.037 (2)	0.032 (2)	0.0064 (17)	0.0060 (15)	0.0045 (17)
C30	0.047 (2)	0.028 (2)	0.0230 (18)	−0.0080 (17)	0.0034 (15)	−0.0012 (15)
C31	0.039 (2)	0.051 (3)	0.034 (2)	0.0018 (18)	0.0027 (16)	−0.0033 (19)
C32	0.046 (2)	0.048 (3)	0.037 (2)	0.0106 (19)	0.0025 (17)	−0.0035 (19)
C33	0.062 (2)	0.026 (2)	0.0265 (19)	0.0013 (18)	0.0015 (17)	0.0006 (16)
C34	0.049 (2)	0.032 (2)	0.033 (2)	0.0055 (18)	0.0071 (16)	0.0002 (17)
C35	0.045 (2)	0.037 (2)	0.035 (2)	0.0054 (18)	0.0081 (16)	−0.0008 (17)
C36	0.0324 (19)	0.031 (2)	0.030 (2)	0.0027 (16)	−0.0089 (15)	0.0029 (16)
C37	0.103 (3)	0.026 (2)	0.042 (2)	0.023 (2)	−0.011 (2)	0.0083 (19)
C38	0.090 (3)	0.040 (3)	0.064 (3)	0.015 (2)	−0.025 (2)	0.010 (2)

### Geometric parameters (Å, °)

**Table:**

Cl1—C14	1.737 (3)	C12—H12	0.9500
Cl2—C33	1.735 (3)	C13—C14	1.385 (5)
S1—C8	1.716 (3)	C13—H13	0.9500
S1—C7	1.721 (3)	C14—C15	1.357 (5)
S2—C27	1.713 (3)	C15—C16	1.397 (4)
S2—C26	1.722 (3)	C15—H15	0.9500
O1—N1	1.236 (3)	C16—H16	0.9500
O2—N1	1.222 (3)	C18—C19	1.499 (4)
O3—C11	1.371 (4)	C18—H18A	0.9900
O3—C10	1.441 (4)	C18—H18B	0.9900
O4—C17	1.329 (4)	C19—H19A	0.9800
O4—C18	1.463 (3)	C19—H19B	0.9800
O5—C17	1.202 (4)	C19—H19C	0.9800
O6—N3	1.229 (3)	C20—C21	1.388 (4)
O7—N3	1.235 (3)	C20—C25	1.408 (4)
O8—C30	1.373 (4)	C20—H20	0.9500
O8—C29	1.430 (4)	C21—C22	1.387 (4)
O9—C36	1.332 (4)	C21—H21	0.9500
O9—C37	1.480 (4)	C22—C23	1.380 (4)
O10—C36	1.198 (4)	C23—C24	1.379 (4)
N1—C3	1.483 (4)	C23—H23	0.9500
N2—C7	1.316 (4)	C24—C25	1.391 (4)
N2—C9	1.368 (4)	C24—H24	0.9500
N3—C22	1.475 (4)	C25—C26	1.479 (4)
N4—C26	1.311 (4)	C27—C28	1.363 (4)
N4—C28	1.368 (4)	C27—C36	1.468 (5)
C1—C2	1.384 (4)	C28—C29	1.510 (4)
C1—C6	1.401 (4)	C29—H29A	0.9900
C1—H1	0.9500	C29—H29B	0.9900
C2—C3	1.384 (4)	C30—C31	1.375 (5)
C2—H2	0.9500	C30—C35	1.389 (4)
C3—C4	1.380 (4)	C31—C32	1.381 (4)
C4—C5	1.378 (4)	C31—H31	0.9500
C4—H4	0.9500	C32—C33	1.391 (5)
C5—C6	1.395 (4)	C32—H32	0.9500
C5—H5	0.9500	C33—C34	1.365 (5)
C6—C7	1.478 (4)	C34—C35	1.402 (4)
C8—C9	1.368 (4)	C34—H34	0.9500
C8—C17	1.468 (4)	C35—H35	0.9500
C9—C10	1.493 (4)	C37—C38	1.420 (5)
C10—H10A	0.9900	C37—H37A	0.9900
C10—H10B	0.9900	C37—H37B	0.9900
C11—C12	1.382 (5)	C38—H38A	0.9800
C11—C16	1.395 (5)	C38—H38B	0.9800
C12—C13	1.373 (4)	C38—H38C	0.9800
			
C8—S1—C7	89.05 (15)	H18A—C18—H18B	108.7
C27—S2—C26	89.03 (15)	C18—C19—H19A	109.5
C11—O3—C10	117.6 (3)	C18—C19—H19B	109.5
C17—O4—C18	116.1 (3)	H19A—C19—H19B	109.5
C30—O8—C29	116.6 (2)	C18—C19—H19C	109.5
C36—O9—C37	113.7 (3)	H19A—C19—H19C	109.5
O2—N1—O1	124.3 (3)	H19B—C19—H19C	109.5
O2—N1—C3	118.3 (3)	C21—C20—C25	120.1 (3)
O1—N1—C3	117.5 (3)	C21—C20—H20	119.9
C7—N2—C9	110.5 (3)	C25—C20—H20	119.9
O6—N3—O7	123.3 (3)	C20—C21—C22	118.0 (3)
O6—N3—C22	118.3 (3)	C20—C21—H21	121.0
O7—N3—C22	118.4 (3)	C22—C21—H21	121.0
C26—N4—C28	110.4 (3)	C23—C22—C21	123.2 (3)
C2—C1—C6	120.1 (3)	C23—C22—N3	118.3 (3)
C2—C1—H1	120.0	C21—C22—N3	118.5 (3)
C6—C1—H1	120.0	C22—C23—C24	118.2 (3)
C1—C2—C3	118.4 (3)	C22—C23—H23	120.9
C1—C2—H2	120.8	C24—C23—H23	120.9
C3—C2—H2	120.8	C23—C24—C25	120.9 (3)
C4—C3—C2	122.7 (3)	C23—C24—H24	119.6
C4—C3—N1	118.3 (3)	C25—C24—H24	119.6
C2—C3—N1	118.9 (3)	C24—C25—C20	119.6 (3)
C5—C4—C3	118.7 (3)	C24—C25—C26	118.8 (3)
C5—C4—H4	120.7	C20—C25—C26	121.6 (3)
C3—C4—H4	120.7	N4—C26—C25	122.0 (3)
C4—C5—C6	120.3 (3)	N4—C26—S2	115.0 (2)
C4—C5—H5	119.8	C25—C26—S2	122.9 (2)
C6—C5—H5	119.8	C28—C27—C36	133.4 (3)
C5—C6—C1	119.8 (3)	C28—C27—S2	110.2 (3)
C5—C6—C7	118.6 (3)	C36—C27—S2	116.3 (2)
C1—C6—C7	121.6 (3)	C27—C28—N4	115.4 (3)
N2—C7—C6	122.3 (3)	C27—C28—C29	127.3 (3)
N2—C7—S1	115.0 (2)	N4—C28—C29	117.2 (3)
C6—C7—S1	122.6 (2)	O8—C29—C28	107.0 (2)
C9—C8—C17	131.6 (3)	O8—C29—H29A	110.3
C9—C8—S1	110.3 (2)	C28—C29—H29A	110.3
C17—C8—S1	118.0 (2)	O8—C29—H29B	110.3
N2—C9—C8	115.1 (3)	C28—C29—H29B	110.3
N2—C9—C10	117.9 (3)	H29A—C29—H29B	108.6
C8—C9—C10	126.9 (3)	O8—C30—C31	116.4 (3)
O3—C10—C9	106.2 (3)	O8—C30—C35	124.2 (3)
O3—C10—H10A	110.5	C31—C30—C35	119.4 (3)
C9—C10—H10A	110.5	C30—C31—C32	120.8 (3)
O3—C10—H10B	110.5	C30—C31—H31	119.6
C9—C10—H10B	110.5	C32—C31—H31	119.6
H10A—C10—H10B	108.7	C31—C32—C33	119.3 (4)
O3—C11—C12	116.3 (3)	C31—C32—H32	120.4
O3—C11—C16	124.0 (3)	C33—C32—H32	120.4
C12—C11—C16	119.7 (3)	C34—C33—C32	121.2 (3)
C13—C12—C11	121.0 (4)	C34—C33—Cl2	120.6 (3)
C13—C12—H12	119.5	C32—C33—Cl2	118.3 (3)
C11—C12—H12	119.5	C33—C34—C35	119.0 (3)
C12—C13—C14	119.1 (4)	C33—C34—H34	120.5
C12—C13—H13	120.4	C35—C34—H34	120.5
C14—C13—H13	120.4	C30—C35—C34	120.4 (3)
C15—C14—C13	120.8 (3)	C30—C35—H35	119.8
C15—C14—Cl1	120.1 (3)	C34—C35—H35	119.8
C13—C14—Cl1	119.1 (3)	O10—C36—O9	123.2 (3)
C14—C15—C16	120.8 (4)	O10—C36—C27	123.2 (3)
C14—C15—H15	119.6	O9—C36—C27	113.7 (3)
C16—C15—H15	119.6	C38—C37—O9	109.6 (3)
C11—C16—C15	118.5 (4)	C38—C37—H37A	109.8
C11—C16—H16	120.7	O9—C37—H37A	109.8
C15—C16—H16	120.7	C38—C37—H37B	109.8
O5—C17—O4	123.8 (3)	O9—C37—H37B	109.8
O5—C17—C8	123.5 (3)	H37A—C37—H37B	108.2
O4—C17—C8	112.7 (3)	C37—C38—H38A	109.5
O4—C18—C19	106.2 (3)	C37—C38—H38B	109.5
O4—C18—H18A	110.5	H38A—C38—H38B	109.5
C19—C18—H18A	110.5	C37—C38—H38C	109.5
O4—C18—H18B	110.5	H38A—C38—H38C	109.5
C19—C18—H18B	110.5	H38B—C38—H38C	109.5
			
C6—C1—C2—C3	0.2 (5)	C25—C20—C21—C22	−0.4 (4)
C1—C2—C3—C4	−0.2 (5)	C20—C21—C22—C23	0.9 (4)
C1—C2—C3—N1	−178.6 (3)	C20—C21—C22—N3	−178.5 (3)
O2—N1—C3—C4	−2.4 (5)	O6—N3—C22—C23	−179.2 (3)
O1—N1—C3—C4	178.1 (3)	O7—N3—C22—C23	−0.5 (4)
O2—N1—C3—C2	176.1 (3)	O6—N3—C22—C21	0.3 (4)
O1—N1—C3—C2	−3.4 (5)	O7—N3—C22—C21	178.9 (3)
C2—C3—C4—C5	0.7 (5)	C21—C22—C23—C24	−0.6 (4)
N1—C3—C4—C5	179.2 (3)	N3—C22—C23—C24	178.7 (3)
C3—C4—C5—C6	−1.3 (5)	C22—C23—C24—C25	−0.1 (4)
C4—C5—C6—C1	1.4 (5)	C23—C24—C25—C20	0.5 (4)
C4—C5—C6—C7	−178.3 (3)	C23—C24—C25—C26	−178.8 (3)
C2—C1—C6—C5	−0.8 (5)	C21—C20—C25—C24	−0.2 (4)
C2—C1—C6—C7	178.9 (3)	C21—C20—C25—C26	179.0 (3)
C9—N2—C7—C6	−179.7 (3)	C28—N4—C26—C25	179.8 (3)
C9—N2—C7—S1	0.2 (4)	C28—N4—C26—S2	−0.9 (3)
C5—C6—C7—N2	2.9 (5)	C24—C25—C26—N4	−1.8 (4)
C1—C6—C7—N2	−176.8 (3)	C20—C25—C26—N4	179.0 (3)
C5—C6—C7—S1	−177.0 (2)	C24—C25—C26—S2	179.0 (2)
C1—C6—C7—S1	3.3 (4)	C20—C25—C26—S2	−0.3 (4)
C8—S1—C7—N2	−0.2 (3)	C27—S2—C26—N4	0.8 (2)
C8—S1—C7—C6	179.7 (3)	C27—S2—C26—C25	−179.9 (3)
C7—S1—C8—C9	0.1 (3)	C26—S2—C27—C28	−0.4 (2)
C7—S1—C8—C17	−176.7 (3)	C26—S2—C27—C36	−177.3 (3)
C7—N2—C9—C8	−0.2 (4)	C36—C27—C28—N4	176.2 (3)
C7—N2—C9—C10	178.7 (3)	S2—C27—C28—N4	0.0 (3)
C17—C8—C9—N2	176.2 (3)	C36—C27—C28—C29	−1.5 (6)
S1—C8—C9—N2	0.0 (4)	S2—C27—C28—C29	−177.6 (2)
C17—C8—C9—C10	−2.6 (6)	C26—N4—C28—C27	0.6 (4)
S1—C8—C9—C10	−178.7 (3)	C26—N4—C28—C29	178.5 (2)
C11—O3—C10—C9	−172.6 (2)	C30—O8—C29—C28	−168.5 (3)
N2—C9—C10—O3	−104.0 (3)	C27—C28—C29—O8	65.7 (4)
C8—C9—C10—O3	74.7 (4)	N4—C28—C29—O8	−111.9 (3)
C10—O3—C11—C12	175.2 (3)	C29—O8—C30—C31	154.8 (3)
C10—O3—C11—C16	−7.1 (4)	C29—O8—C30—C35	−26.7 (5)
O3—C11—C12—C13	177.7 (3)	O8—C30—C31—C32	176.5 (3)
C16—C11—C12—C13	−0.1 (5)	C35—C30—C31—C32	−2.1 (6)
C11—C12—C13—C14	−0.4 (5)	C30—C31—C32—C33	0.5 (6)
C12—C13—C14—C15	−0.1 (5)	C31—C32—C33—C34	1.3 (6)
C12—C13—C14—Cl1	180.0 (3)	C31—C32—C33—Cl2	−179.2 (3)
C13—C14—C15—C16	1.0 (6)	C32—C33—C34—C35	−1.5 (6)
Cl1—C14—C15—C16	−179.1 (3)	Cl2—C33—C34—C35	179.0 (3)
O3—C11—C16—C15	−176.6 (3)	O8—C30—C35—C34	−176.6 (3)
C12—C11—C16—C15	1.0 (5)	C31—C30—C35—C34	1.8 (5)
C14—C15—C16—C11	−1.5 (5)	C33—C34—C35—C30	0.0 (5)
C18—O4—C17—O5	2.2 (5)	C37—O9—C36—O10	2.9 (5)
C18—O4—C17—C8	−176.8 (3)	C37—O9—C36—C27	−175.8 (3)
C9—C8—C17—O5	−172.4 (4)	C28—C27—C36—O10	−167.3 (3)
S1—C8—C17—O5	3.5 (5)	S2—C27—C36—O10	8.7 (5)
C9—C8—C17—O4	6.6 (5)	C28—C27—C36—O9	11.4 (5)
S1—C8—C17—O4	−177.5 (2)	S2—C27—C36—O9	−172.6 (2)
C17—O4—C18—C19	174.1 (3)	C36—O9—C37—C38	169.9 (3)

### Hydrogen-bond geometry (Å, °)

**Table:**

*D*—H···*A*	*D*—H	H···*A*	*D*···*A*	*D*—H···*A*
C1—H1···O5^i^	0.95	2.37	3.314 (4)	172
C20—H20···O10^ii^	0.95	2.50	3.450 (4)	173

Symmetry codes: (i) −*x*+1, −*y*+1, −*z*+1; (ii) −*x*+1, −*y*+1, −*z*.
